# Spontaneous eye movements reflect the representational geometries of conceptual spaces

**DOI:** 10.1073/pnas.2403858121

**Published:** 2024-04-18

**Authors:** Simone Viganò, Rena Bayramova, Christian F. Doeller, Roberto Bottini

**Affiliations:** ^a^Max Planck Institute for Human Cognitive and Brain Sciences, Department of Psychology, Leipzig 04103, Germany; ^b^Center for Mind/Brain Sciences, University of Trento, Rovereto 38068, Italy; ^c^Max Planck School of Cognition, Max Planck Institute of Human Cognitive and Brain Sciences, Department of Psychology, Leipzig 04103, Germany; ^d^Kavli Institute for Systems Neuroscience, Center for Neural Computation, The Egil and Pauline Braathen and Fred Kavli Center for Cortical Microcircuits, Jebsen Center for Alzheimer’s Disease, Norwegian University of Science and Technology, Trondheim 7491, Norway

**Keywords:** eye movements, representational geometry, conceptual spaces, cognitive map

## Abstract

Functional neuroimaging studies indicate that the human brain can represent concepts and their relational structure in memory using coding schemes typical of spatial navigation. However, whether we can read out the internal representational geometries of conceptual spaces solely from human behavior remains unclear. Here, we report that the relational structure between concepts in memory might be reflected in spontaneous eye movements during verbal fluency tasks: When we asked participants to randomly generate numbers, their eye movements correlated with distances along the left-to-right one-dimensional geometry of the number space (mental number line), while they scaled with distance along the ring-like two-dimensional geometry of the color space (color wheel) when they randomly generated color names. Moreover, when participants randomly produced animal names, eye movements correlated with low-dimensional similarity in word frequencies. These results suggest that the representational geometries used to internally organize conceptual spaces might be read out from gaze behavior.

## Significance

Understanding how humans represent concepts and their relations is a crucial question in cognitive (neuro)science. By analyzing eye movements during verbal fluency tasks, we observed that people directed their gaze to the left or right before mentioning a small or large number, while they directed it to a closer or further position in bi-dimensional visual coordinates when mentioning colors that were, respectively, similar or dissimilar in the color wheel. These results suggested eye movements as a potential behavioral readout of low-dimensional cognitive maps of concepts, thus we investigated participants’ gaze behavior when randomly generating animal names, observing that, in this case, spontaneous gaze fixations reflected similarity in word frequency along a left-to-right axis.

When humans search for information in memory, they partially repurpose the neuronal mechanisms that evolved for navigating and interacting with the surrounding environment (1–5). For instance, the hippocampal-entorhinal system, known for its role in spatial mapping and orientation (see e.g., refs. 6 and 7), is also recruited to represent the relational structure between items in memory as they were points of internal “cognitive maps” (8–13). However, how such representations directly translate into behavior is less clear. Can we read out these representational geometries of conceptual spaces from human behavior?

Evidence from separate research lines converges in suggesting gaze behavior as a good candidate for investigating this question (for reviews see refs. 14–16). Monkey electrophysiology, for instance, shows that neurons in the hippocampal formation not only respond to locations in space, but also map the visual field both during visual tasks and free exploration of the environment (15, 17–22). Human functional neuroimaging confirms and extends the notion that hippocampal activity is linked to eye movements and gaze behavior in a variety of tasks spanning for instance from visual tracking (23), visual search (24), and relational memory (25). Consistently, neuropsychology reveals that amnesic patients with hippocampal damage search in the visual environment less efficiently with their eyes (26–28). Therefore, given i) the role that hippocampal cognitive maps play for concept representation and mental search and ii) the link that exists between gaze behavior and hippocampal activity, we hypothesized that eye movements might reflect the relational structure of concepts in memory, and that they can be used as a behavioral readout of low dimensional cognitive maps.

Preliminary evidence for this view is offered by studies in the fields of numerical cognition and time representation. In Western cultures, both time and numbers are known to be mentally represented along one-dimensional (1D) structures [“mental time line” (29, 30) and “mental number line” (31)] mostly oriented from left to right. Consistent with this representation, Loetscher et al. (32) showed that when participants randomly generate numbers as they come to their mind, they move their eyes to either the left or the right if the next number is respectively smaller or larger compared to the previous one. This effect has been extended to arithmetic problems, comparing addition (associated to the right/up) and subtraction (associated to the left/down) (33–35). Similarly, when people encode, recall, or recognize events that happened either in the past or in the future, they look down/to the left for the former, and up/to the right for the latter (36–38).

**Fig. 1.**
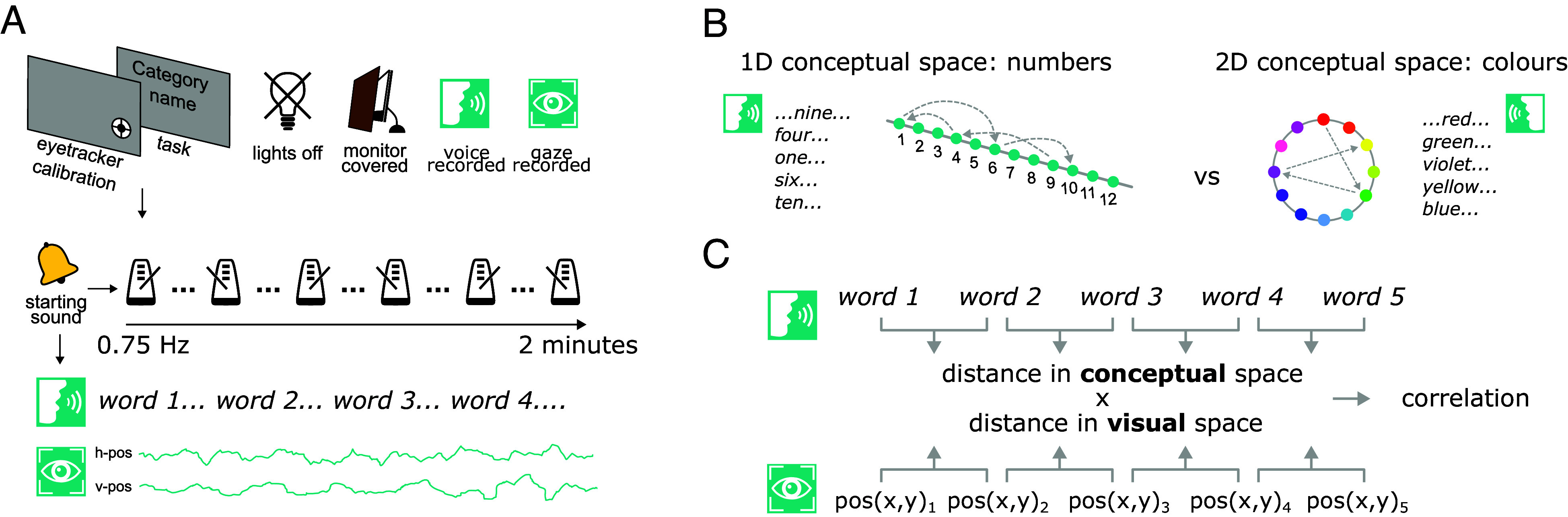
Tracking eye movements during mental search. (*A*) Participants sat in the dark in front of a desktop computer wearing earplugs, with their eye movements monitored via an eye tracker. They started each block with an eye-tracking calibration, followed by visually displayed instructions: They were asked to generate, after a starting sound and in a random order, numbers (from 1 to 12) or 12 color names (red, orange, yellow, lime, green, turquoise, cyan, blue, violet, lilac, pink, and magenta), by speaking into a microphone. After this, the experimenter covered the PC monitor with a wooden panel to block any remaining source of light and potential visual interference. Then, participants were alerted by a starting sound, followed by the sound of a metronome, presented via earphones, at a frequency of 0.75 Hz, introduced to engage participants in generating words. Each block lasted 2 min and there were three blocks per conceptual domain. (*B*) The number space and the color space have two distinctive geometries, namely a 1D number line and a 2D color ring (or “wheel,” where distance between colors scales with their conceptual/perceptual similarity forming a canonical color wheel, as revealed by multidimensional scaling applied to similarity judgments, ref. 41). We hypothesized that eye movements during random number vs. color generation should reflect the representational geometry of the respective underlying conceptual space. (*C*) To test our hypothesis, we applied a correlational analysis approach. The main analysis, following Loetscher et al. (32), consisted in the extraction of the trial-by-trial distance from one concept to the other in the underlying conceptual space (e.g., number “four” followed by “ten,” or color “red” followed by “blue”) and in its correlation with trial-by-trial gaze displacement. Additional analyses are described in *Results* and *Methods*.

These results suggest that spontaneous gaze behavior might reflect the internal relational structure of the mental spaces where concepts are sampled from, but the existent evidence is limited to highly conventionalized, culturally specific, and unidimensional representation of order (3). To support the hypothesis of a generalized mechanism of how concept relations are reflected in oculomotor behavior, it is important to collect and compare evidence from conceptual domains with distinct representational geometries. To this end, we analyzed spontaneous eye movements when 30 adult participants randomly generated i) numbers, known to be arranged in a 1D structure that in Western cultures is usually oriented from left to right, and ii) colors, known to have a psychological two-dimensional (2D) structure akin to the “color wheel” or “ring” (39), typically observed through similarity judgments; see e.g., refs. 40 and 41; see Fig. 1 *A* and *B*. If our hypothesis is correct and gaze behavior can reflect the representational geometry of conceptual spaces, then eye movements should correlate with the particular relational structures of the two conceptual domains: the left-to-right 1D arrangement of the mental number line in the case of numbers and the 2D ring-like structure of the color wheel in the case of colors (Fig. 1 *B* and *C*).

## Results

### Spontaneous Eye Movements Reflect the 1D Relational Structure of the Number Space during Mental Search.

Participants easily completed the “number” condition, closely following the rhythm of the metronome and randomly sampling the 12 numbers with roughly the expected frequency (*SI Appendix*, Fig. S1). For each number word, we extracted the median gaze position along the horizontal and vertical axes separately in the 500 ms interval before it was named (*Methods*). Next, we computed a vector with the trial-by-trial numerical difference, by subtracting from the next mentioned number the current one: A negative or positive value would indicate that the following number was smaller or larger, respectively, and the absolute value indicated the magnitude of their difference - that is, their distance along the mental number line. Similarly, we computed a vector of the trial-by-trial difference in gaze position, along the horizontal and vertical axes separately, as well as their combined Euclidean distance in the 2D visual space. For instance, a negative or positive value in the vector representing change of position along the horizontal axis would indicate that the subject had moved the eyes towards the left or the right, respectively, compared to the previous trial, and the absolute value would indicate the magnitude of this movement.

**Fig. 2.**
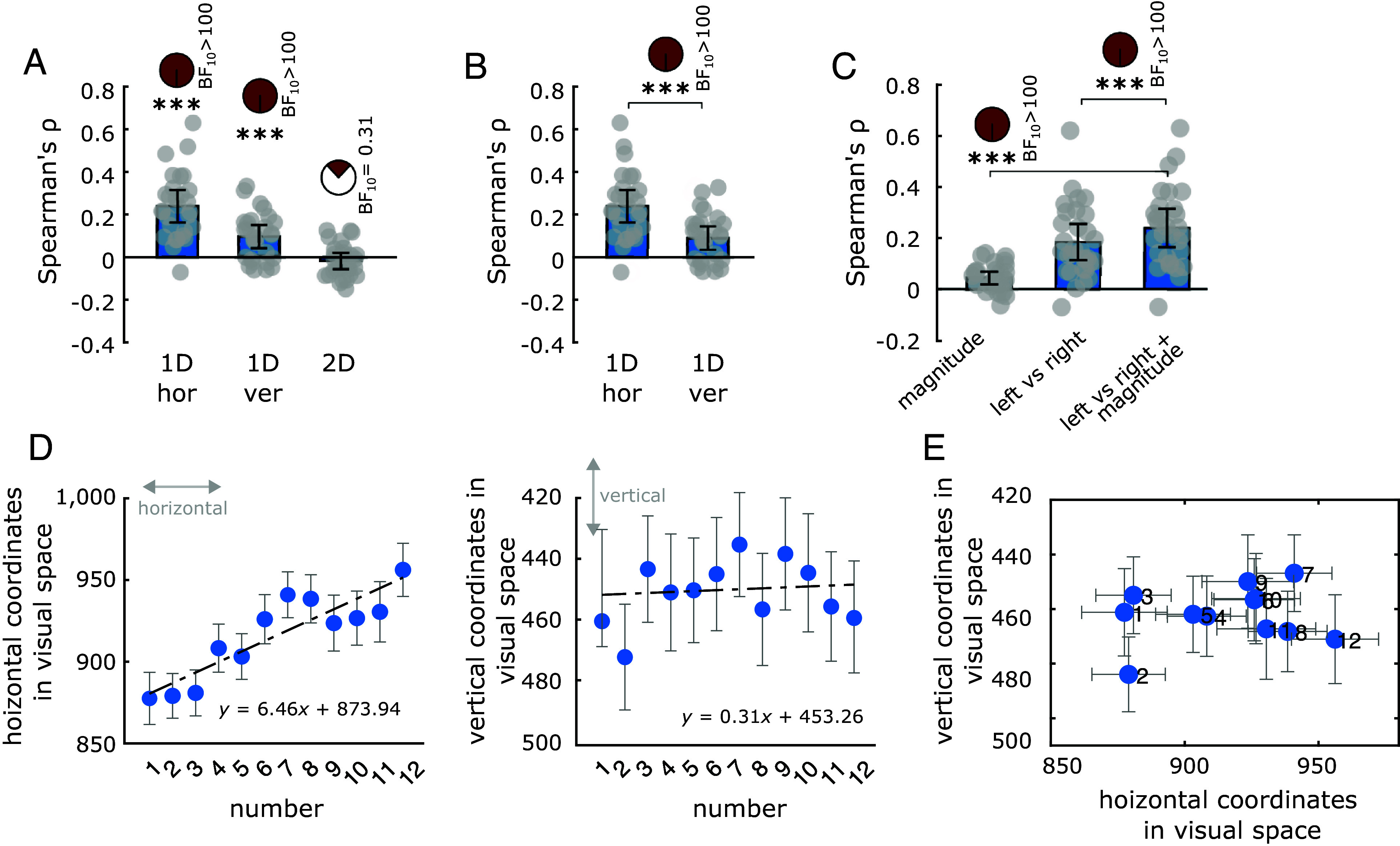
Eye movements reflect the 1D, horizontal structure of the number space. (*A*) Fisher-transformed correlation coefficients between eye movements and signed change in generated numbers. 1D hor = horizontal eye movements (left–right), 1D ver = vertical eye movements (up–down), 2D = bidimensional eye movements (2D Euclidean distance). Bars indicate SEM. (*B*) Comparing the correlations with horizontal and vertical eye movements with respect to the zero, to evaluate whether one of the two better reflects the underlying representational geometry of numbers. Data are the same as plot *a*, here isolated for visualization purposes. (*C*) Comparing the correlation with eye movements when only absolute magnitude of the number change is considered (*Left* bar) vs. when only increase/decrease in numerosity is taken into account (*Middle* bar) vs. their combination (*Right* bar). (*D*) Absolute horizontal (*Left* bar) and vertical (*Right* bar) gaze coordinates before generating each number, averaged across participants. Error bars indicate SEM. For the vertical dimension, the y-axis is reversed in order as the eye-tracker considers the origin 0 at the top of the screen (*E*) visualization of the average gaze position for each number in visual space. ***P < 0.001; BF_10_ = Bayes Factor in favor of H1 over H0.

We observed a significant correlation between the signed difference in generated numbers and the signed change in gaze position along both the horizontal [left–right; mean ρ = 0.24, SD = 0.15, t = 8.43, *P* < 0.001 (*t* test), Bayes Factor in favor of H1 over H0 (BF_10_) > 100] and the vertical [up–down; mean ρ = 0.09, SD = 0.11, t = 4.43, *P* < 0.001 (*t* test), BF_10_> 100] axes separately, but not for their conjunctive bidimensional displacement in the visual space (mean ρ = −0.02, SD = 0.08, t = −1.19, *P* = 0.24 (*t* test), BF_10_ = 0.31] (Fig. 2*A* and *SI Appendix*, Fig. S2). The effect along the horizontal axis was significantly stronger (relative to zero) than that along the vertical axis [t = 5.05, *P* < 0.001 (paired *t* test), BF_10_ > 100], see Fig. 2*B*, indicating the higher relevance of the horizontal dimension.

Was this effect mostly driven by the unsigned magnitude of the number change (where a change of, for instance, +5 or −5 would be considered the same irrespective of the sign), by its left vs. right direction (where a change of +5 or +7 would be considered the same irrespective of the magnitude), or by their combination? First, we isolated the contribution of magnitude by ignoring both the left vs. right change in eye movements and the signed difference in number change, thus focusing only on the correlation between unsigned magnitudes. We observed a significant effect at the group level [mean ρ = 0.04, SD = 0.05, t = 4.8, *P* < 0.001 (*t* test), BF_10_ > 100]. Second, we isolated the contribution of left vs. right direction by ignoring the magnitude of the number change and that of the eye movements, simply correlating the sign of the number change (plus or minus) with the sign of the change in gaze coordinates. Also in this case we observed a significant effect at the group level [mean ρ = 0.18, SD = 0.14, t = 7.1, *P* < 0.001 (*t* test), BF_10_ > 100]. The former effect was significantly weaker than the latter [t = 5.67, *P* < 0.001 (paired *t* test), BF_10_ > 100], but both correlation scores were significantly lower, at the group level, compared to their combination (both *P* < 0.001 and BF_10_ > 100), thus indicating that although the left vs. right coding contributed more than unsigned magnitude, eye movements best correlated with their combination, which reflected the magnitude of change to the left vs. to the right). These results are summarized in Fig. 2*C*. Could this effect reflect the transition probabilities between numbers (see examples in *SI Appendix*, Fig. S3), with small eye movements being associated for instance with more frequent transitions and large eye movements with less frequent transitions (or vice versa)? This seems to be unlikely, since eye movements did not correlate with these factors [all mean ρ < 0.0004 and *P* > 0.97 (*t* test)].

Next, we looked at individual trials, moving away from trial-by-trial differences and looking at the “absolute” gaze coordinates before a given number was mentioned (e.g., where participants were looking at every time they said “four”). We observed that the smaller the number the participants were about to say, the more they were fixating the left of their visual field, while the bigger the number, the more they were fixating the right [average β of the slope = 6.46, t = 4.26, *P* < 0.001 (*t* test against 0); intercept = 873.94; see Fig. 2 *D*, *Left*]. This effect was statistically absent for the up/down vertical positions [average β of the slope = 0.31, t = 0.57, *P* = 0.57 (*t* test against 0); intercept = 453.26; see Fig. 2 *D*, *Right*], confirming the higher importance of the 1D horizontal axis. Combining the two sets of coordinates allowed us to reconstruct the average gaze position for each number across participants that, despite some expected variability, reflected the mental number line (Fig. 2*E*). The small numbers from 1 to 6 are on average more on the left than large numbers from 7 to 12, which are positioned more on the right.

Finally, we corroborated our findings by applying a decoding approach, where we attempted to classify numbers as small (1 to 6) vs. large (7 to 12) using a linear discriminant analysis (LDA) and a leave-one-out-cross-validated scheme (*Methods*) at the individual subject level. Indeed, a single-trial cross-validated measure would strengthen our conclusion and allow us to test the emergence of the effect in single subjects. Results, significant at the group level [average accuracy = 0.529, SD = 0.058; *P*-value = 0.0134 for a one-sample *t* test against theoretical chance level of 0.5], indicated that this was indeed possible in eight participants, where the observed accuracy of the classifier exceeded with a probability *P* < 0.05 those obtained by randomly shuffling labels 1,000 times (*SI Appendix*, Fig. S4).

In short, when participants spontaneously generated numbers, they moved their eyes according to the 1D, horizontal, and left-to-right oriented, mental number line, consistent with previous findings (32). Having confirmed that with our analytical approach we can recover the underlying geometry of the number space, we then applied it to the color domain.

### Spontaneous Eye Movements Reflect the 2D Relational Structure of the Color Space during Mental Search for Colors.

**Fig. 3.**
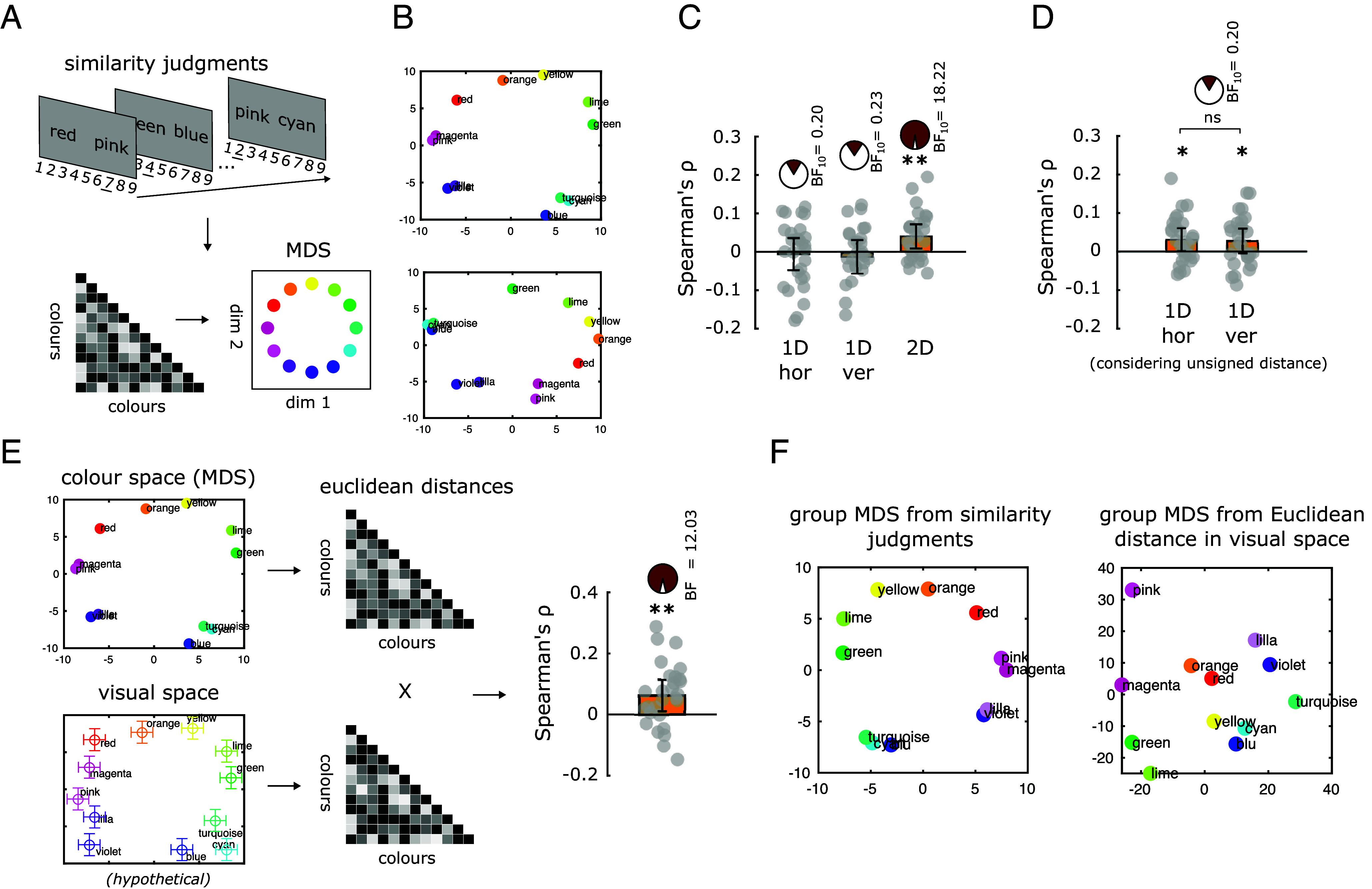
Eye movements reflect the 2D circular structure of color space. (*A*) Similarity judgment task, during which participants evaluate the similarity between two colors (presented with their names) along a scale from 1 to 9 (9 = max. similarity). These pairwise judgments were used to construct a dissimilarity matrix, which is then used to reconstruct the 2D color space using MDS (as in ref. 40). Both the training tasks (*SI Appendix*, Fig. S5) and the similarity judgment task were administered the day before the experiment and on the day of the experiment (*Methods*). Similarity judgments were averaged across the 2 d to increase reliability. (*B*) Examples of the subject-specific color wheel reconstructed with MDS on the first two participants. (*C*) Fisher-transformed correlation coefficients between eye movements and color distance in subject-specific MDS. 1D hor = horizontal eye movements (signed), 1D ver = vertical eye movements (signed). 2D = bidimensional eye movements (2D Euclidean distance). Bars indicate SEM. (*D*) Correlation scores for the horizontal and vertical eye movements after ignoring the signed difference, that is left vs. right. (*E*) Euclidean distances between each color in the subject-specific two dimensional color space (*Top*, real data) and Euclidean distances between the average coordinates for each color the subject-specific 2D visual space (*Bottom*, here shown as a hypothetical circular array). (*F*) Average MDS reconstruction of color space from similarity judgments (*Left*) or from distances in visual space (*Right*) **P < 0.01; BF_10_ = Bayes Factor in favor of H1 over H0.

For the color condition, we asked participants to randomly generate 12 colors that we presented the day before the eye-tracking experiment during an online training session. In this online part, we first showed participants 12 colored patches with their associated color name (*SI Appendix*, Fig. S5*A*), then asked them to recover the correct patch-name association (*SI Appendix*, Fig. S5*B*), and finally asked them to judge the pairwise similarity between the colors on a scale from 1 to 9 (Fig. 3*A*). This training procedure was repeated on the day of the eye-tracking experiment and had two specific purposes: first, to ensure that participants were randomly sampling from comparable color spaces, “foraging” the same 12 color names; second, to collect similarity judgments from each participant to recover, using multidimensional scaling (MDS) as indicated in the work by Shepard and Cooper (40), the bidimensional representation of the color space (the so-called “color-wheel”) for each individual (see examples in Fig. 3*B* and *SI Appendix*, Fig. S6). According to our hypothesis, eye movements should reflect subject-specific distances between colors in the 2D conceptual space.

On the day of the experiment, during the main task, participants performed a random color generation task (similar to the number condition; *SI Appendix*, Fig. S7). Then, we closely followed the same correlational approach that we used for the number blocks (Fig. 1*C*), but now we computed the trial-by-trial change between subsequently pronounced colors by looking at their Euclidean distance in the subject-specific bidimensional MDS (Fig. 3*B*). We observed that the semantic distance between colors was significantly correlated with the bidimensional displacement of gaze position in the visual field [mean ρ = 0.04, SD = 0.06, t = 3.42, *P* = 0.002 (*t* test), BF_10_ = 18.22]: If participants were about to mention a color that was more distant than the previous one in their own MDS-reconstructed color space, they moved their eyes to a more distant position, compared to when the two colors were closer to each other (that is, similar) (Fig. 3*C* and *SI Appendix*, Fig. S8). We did not find a significant correlation when we considered either the horizontal [left–right; mean ρ = −0.006, SD = 0.08, t = −0.36, *P* = 0.72 (*t* test), BF_10_ = 0.20] or the vertical [up–down; mean ρ = −0.009, SD = 0.08, t = −0.57, *P* = 0.57 (*t* test), BF_10_ = 0.23] signed gaze displacement, thus indicating that the specific gaze behavior that predicted color generation was substantially different from the one employed during the number condition (that was sensitive to left–right distinctions). However, the correlation was significantly positive (although to a lower extent) when we considered the unsigned absolute value of unidimensional gaze displacement [horizontal movements: mean ρ = 0.03, t = 2.73, *P* = 0.01 (*t* test), BF_10_ = 4.29; vertical movements: mean ρ = 0.027, t = 2.26, *P* = 0.03 (*t* test), BF_10_ = 1.78; both uncorrected], as expected from the correlation with bidimensional eye movements (Fig. 3*D*). In other words, while the distinction between left and right seems to matter for representing numbers, it is no longer relevant in the case of colors, as expected from the fact that the color wheel has no typical left-vs.-right association. Interestingly, and confirming the difference from the number condition, there was no significant difference between the correlation values for horizontal vs. vertical movements [t = 0.27, *P* = 0.78 (paired *t* test), BF_10_ = 0.2], indicating that in this case both dimensions equally contributed to reflect the representational geometry of the conceptual space (Fig. 3*D*). Could this effect be explained by the transition frequencies from one color to another (see examples in *SI Appendix*, Fig. S9)? As it happened for numbers, this was not the case, because eye movements did not correlate with transition frequencies (all *P* < 0.19).

Next, as in the case of the number condition, we examined whether participants were fixating specific dedicated portions of their visual field before generating a given color. More specifically, we asked whether the median fixation point before saying a color, averaged across trials, was predictive of the position of that color in the color space. To answer this question, we computed two distance matrices. The first one was the distance matrix of pairwise Euclidean distances between colors in the MDS-reconstructed color space (the one used for our previous trial-by-trial analysis) (Fig. 3 *E*, *Top*). The second one was the distance matrix of pairwise Euclidean distances between fixations in the visual field (Fig. 3 *E*, *Bottom*). Across participants, these two matrices were significantly correlated (mean ρ = 0.06, SD = 0.10, t = 3.23, *P* = 0.003, BF_10_ = 12.03; Fig. 3 *E*, *Right*), indicating that the fixation points on the visual field were constrained, at least partially, by the bidimensional arrangement of colors reconstructed through similarity judgments. In other words, the relative position of colors in the bidimensional color space was preserved in gaze fixations. Although there was high variability across participants in their color-specific gaze coordinates (that is, different participants tended to fixate different portions of the visual space for the same color), pairwise relative distances between color-specific fixation points allowed us to reconstruct the approximation of a bidimensional color space at the group level based only on gaze behavior, capturing most of the similarity relations (e.g., in Fig. 3 *F*, *Right*, starting from red-orange and moving clockwise, we observe lilac-violet, then the triplet blue-cyan-turquoise, then green-lime, similar to Fig. 3 *F*, *Left*. Magenta, yellow, and pink, however, do not follow the expected circular arrangement).

To finally corroborate our findings we applied the same decoding approach used in the number condition, now adapted to the situation of colors (*Methods*). We did not find a significant effect in the original dataset, with the classifier performing relatively well in the training dataset (accuracies >55%) but very poorly in the test dataset (accuracy often <50%), indicating potential overfitting that we ascribed to the low number of trials. In order to overcome these shortcomings, we analyzed an additional dataset from a different experiment in which we tested five new subjects using a dense sampling approach mutated by precision neuroimaging (e.g., see refs. 42 and 43). The five participants performed the color foraging task with the same 12 colors of experiment 1, for eight runs/blocks per day, for 3 d in a row, resulting in 24 runs/blocks (compared to only three in experiment 1) and more than 2,000 trials/observations per subject (*Methods*). We applied the same decoding classification procedure used for numbers but now to all the pairwise colors (*Methods*) and in all five subjects the classifier reached an accuracy above chance, with three of them having accuracy higher (*P* < 0.05) than that obtained by 1,000 random permutations of labels (*SI Appendix*, Fig. S10*A*). Interestingly, and in line with our predictions, in all of them, we observed a general positive increase of the accuracy of the classifier as a function of distance between colors (*SI Appendix*, Fig. S10*B*), indicating that the average gaze position associated with two given colors became more distinguishable the more the colors were different. To summarize, we observed that when participants were asked to randomly sample a color space in our experiment (in a dark room where no visual information is available), their eye movements can reflect the bidimensional distances existing between colors on the color wheel.

### Using Eye Movements to Investigate How People Mentally Represent Multidimensional Conceptual Spaces.

Our results showed that spontaneous eye movements during mental search might contain information about the representational geometry of two conceptual spaces, that of numbers and that of colors. Can we use eye movements to read out the representational geometry of multidimensional conceptual spaces that have more complex or unknown underlying structures?

In order to provide a tentative preliminary answer to this question, we focused on the conceptual domain of animals (*SI Appendix*, Fig. S11), which could be organized according to many dimensions, such as taxonomy, size, speed, dangerousness, starting letter, frequency in a language, and so on. Is the relationship between concepts in one or more of these dimensions reflected in eye movements?

**Fig. 4.**
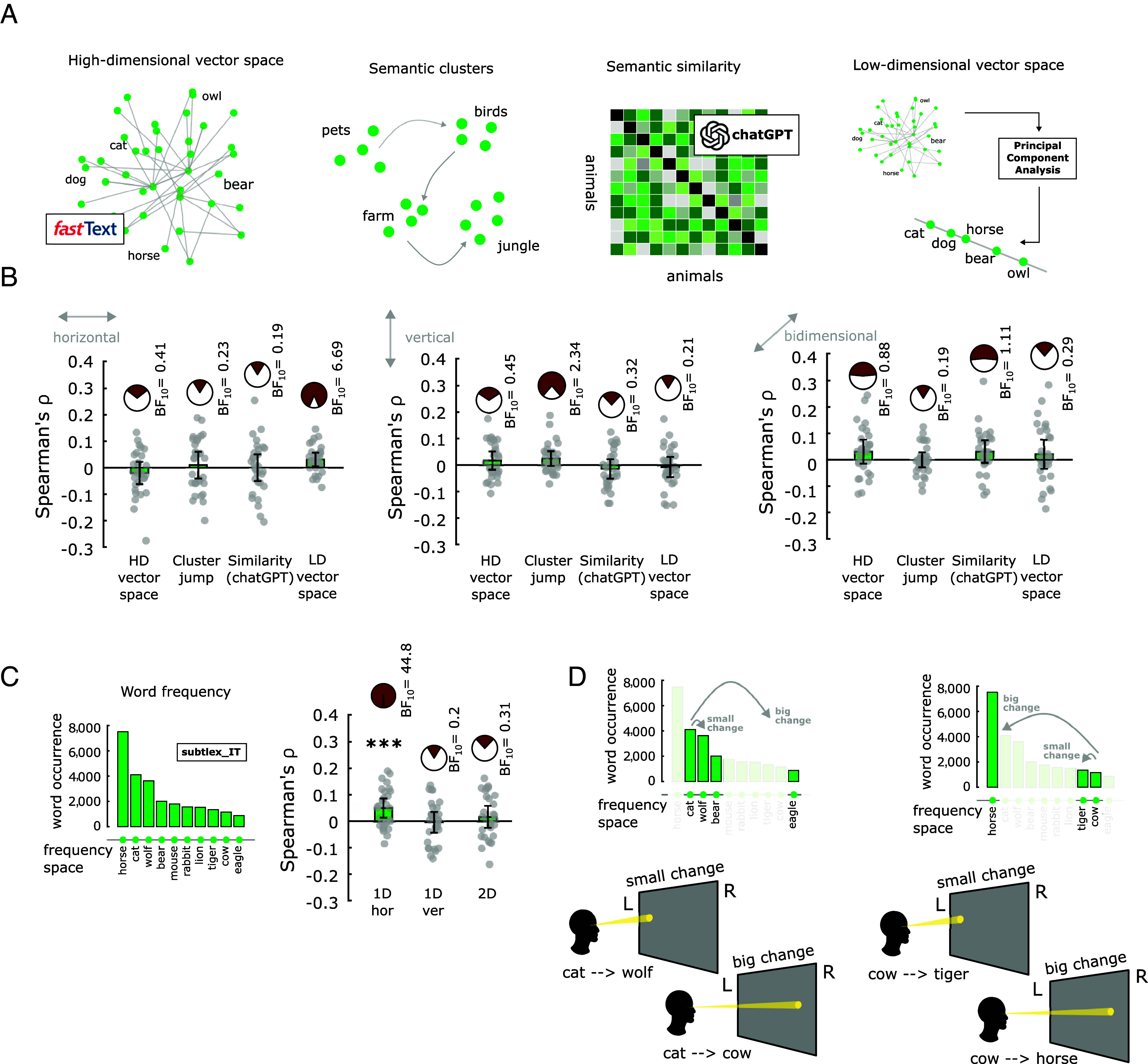
Eye movements reflect the low-dimensional projection of the high-dimensional animal space. (*A*) Distances between animal words were modeled i) as cosine distances between high-dimensional linguistic vectors extracted from fastText; ii) as jumps across semantic clusters; iii) as the inverse of semantic similarity as judged by chatGPT; iv) as Euclidean distances along a low-dimensional projection (PC1) of fastText vectors. (*B*) Correlation between horizontal (*Top*), vertical (*Middle*), and bidimensional (*Bottom*) gaze displacement and the four modeled distances. (*C*) Modeling distances between animal words as differences in their linguistic frequencies (*Left*) and correlating them with patterns of eye movements (*Right*); (*D*) Schematic representation of the only significant effect: Participants look more to the left if they are about to mention an animal that has a small difference in frequency in Italian compared to the previous one, while they look more to the right if they are about to mention an animal that has a large difference in frequency in Italian compared to the previous one.

To examine this, we followed the same correlational approach used above (Fig. 1*C*), but now we modeled distances between animal names in the following ways: i) as their cosine distance between animal word vectors extracted from a distributional language model (word2vec FastText, (44)), which has been shown to be a reliable model of high-dimensional semantic representations in the human mind (e.g., refs. 45–48) (Fig. 4 *A*, *Left*); ii) as change in semantic clusters, because it was shown that when human participants randomly produce animal names during semantic foraging, they adopt a local-to-global search strategy in which they tend to produce animals belonging to the same clusters (e.g., farm animals) before moving to other ones (e.g., birds) (49–51) (Fig. 4 *A*, *Middle-Left*); and iii) as human-like semantic similarity judgments recovered from a state-of-the-art large language model (chatGPT, see *Methods* and *SI Appendix*, Fig. S12), which has shown human-like performance in a variety of tasks (52, 53) (Fig. 4 *A*, *Middle-Right*). Although these approaches have been proposed to map human cognition in various ways, none of them significantly correlated with neither horizontal nor vertical eye movements, nor with their bidimensional combination (all *P*-values > 0.10 uncorrected; Fig. 4*B*).

An alternative possibility, then, is that eye movements reflect lower-dimensional geometries between animal concepts. Previous studies indeed found that low-dimensional projections (through Principal Component Analysis, PCA) of linguistic vectors contain relevant semantic information (e.g., ref. 54) and correlate with human hippocampal activity during memory tasks (e.g., ref. 55). Thus, we computed distances between animal words as their Euclidean distances on the first principal component (PC1) of FastText space, and we observed a significant correlation with horizontal eye movements (mean ρ = 0.03, SD = 0.05, t = 2.29, *P* = 0.006, BF_10_ = 6.69) (Fig. 4*B*): Participants looked more to the left when they were about to say an animal that was similar/closer compared to the previous one, and more to the right when they were about to say an animal that was different/distant compared to the previous one. In addition, Hollis and Westbury (54) proposed that PC1 of linguistic vectors could be interpreted as representing, at least partially, word frequency. Indeed, consistent with this idea, we found that horizontal eye movements significantly correlated with differences in word frequency as computed by Crepaldi et al. (56) (mean ρ = 0.05, SD = 0.07, t = 3.86, *P* < 0.001, BF_10_ = 44.8). This result is statistically significant also when considering the high number of multiple comparisons we are performing in this exploratory analysis, which would lead to an alpha value of 0.05/15 = 0.003 according to Bonferroni correction) (Fig. 4*C*). Confirming Hollis and Westbury (54) intuition, distances on PC1 and differences in word frequencies were correlated with each other (ρ = 0.3, *P* < 0.001), thus motivating the use of a partial correlation approach to isolate the real contribution of the two factors to the correlation with eye movements. The results indicate that the effect with distances along PC1 was no longer significant after controlling for difference in word frequency (*P* = 0.14, BF_10_ = 0.54), therefore among the tested models, eye movements best reflected distance in “frequency space.” More specifically, and interestingly, the results indicate that participants looked more to the left before mentioning an animal that has a similar frequency value compared to the previous one, and they looked more to the right before mentioning an animal that has a different frequency value compared to the previous one (Fig. 4*D*). This effect was not driven by movements on one side of the space only, because small distances were biased toward the left as much as large distances were toward the right (*P* > 0.22).

## Discussion

According to the cognitive map theory (57, 58) and more recent accounts (2, 3, 5), the neurocognitive machinery that supports spatial orientation in mammals can also be recruited to represent more abstract conceptual spaces, suggesting common underlying representational mechanisms for spatial and nonspatial knowledge. While most of the empirical support to this proposal comes from neuroimaging, there has been much less evidence on the behavioral level. In light of the existing link between gaze behavior, navigation, and hippocampal formation (see ref. 16 for a review), we hypothesized that human eye movements might reflect the representational geometries of conceptual spaces, at least during specific tasks and situations.

In this study, we reported preliminary evidence in support of this hypothesis. First, when participants randomly generated numbers, their horizontal eye movements correlated with the left-to-right 1D arrangement of the “mental number line.” Second, when they randomly generated colors, their eye movements correlated with the ring-like 2D arrangement of the “color wheel.” Third, we showed that information conveyed by eye movements can then be used to get insights on how people mentally organize multidimensional conceptual spaces during free mental search: We collected preliminary evidence that when randomly producing animal names, horizontal eye movements reflected similarity in word frequency.

Our results are in line with a large body of work indicating that eye movements reflect more than externally driven visual search behavior (e.g., scan a visual scene to find a specific target). They can reveal critical information about our internal mental processes, being involved in memory (59), imagination (60), attention (61), numerical and arithmetic cognition (32–34, 62), time representation (36, 63), working memory (64) and reasoning (65). Here, we showed that they also reflect the internal task-relevant relational structure (or representational geometry) of conceptual spaces. Interestingly, we observed this effect by analyzing a time window that preceded the generation of concept words during a verbal fluency task (500 ms before word onset), and this speaks in favor of the proposal that eye movements might even precede conscious recollection (14), potentially guiding memory expression toward action (66).

Some additional aspects demand particular attention. Are these gaze biases causally involved in mental search, or do they represent an epiphenomenal consequence of our internal strategies for mental search? A recent study addressed the question by asking participants to solve addition problems while following a moving dot with their eyes (67). The author showed that performance was faster for upward movements compared to downward or free movements, suggesting that the shift of gaze might not be a simple epiphenomenon. However, this evidence is limited to a 1D numerical/mathematical space, and recent studies have shown that grid-like coding during visual search, in monkeys and humans, emerged also while keeping fixation and searching the visual space with covert attention (68, 69). Further experiments are needed to better characterize the relationship between eye movements, attention, and internal low-dimensional cognitive maps, across different conceptual spaces and tasks.

What is the relationship between eye movements and brain activity? What are the neural mechanisms that are linked to the gaze biases observed in the present study and their representation of conceptual geometries? While the hippocampus has been related to eye movements in a variety of tasks and contexts (17, 23, 25), regions in frontal and parietal lobes are well known for their role in gaze control (70). The parietal cortex, in particular, has recently been proposed as a potential region for representing conceptual knowledge using spatial codes related to egocentric reference frames (3), which would be complementary to the allocentric cognitive maps that emerge in the hippocampal formation (see ref. 2). Indeed, recent empirical evidence indicates that when participants are engaged in goal-directed “conceptual navigation,” mentally searching for specific goal stimuli in their mind, their parietal cortex represents the navigated conceptual space using egocentric-like codes (71). Crucially, this was associated with concurrent activation of the oculomotor system, which kept track of goal position in conceptual space from a first-person perspective. The results reported in the current study are consistent with this previous evidence as well as, potentially, with the proposal of a specific role of the parietal cortex in representing structural knowledge (72–74), specially for the number domain (33). Future studies should address this point more directly, leveraging the concurrent use of eye-tracking technologies with whole brain neuroimaging.

Finally, an important finding of our experiments was the observation that when participants were engaged in a task that required them to sample concepts from a multidimensional space—that of animals—eye movements correlated with a rather simple low-dimensional projection of that space, representing word frequencies. This potentially resonates with recent findings that visual exploration dynamics are intrinsically low-dimensional (75), but whether this can be extended to all conceptual domains or, crucially, all task situations, remains unclear: Word frequency is a linguistic feature that was particularly useful in our verbal fluency task, but should eye movements correlate with other dimensions if the task imposes more stringent constraints is an open question. Similarly, it could be the case that other dimensions or models not tested in our experiment could correlate better with eye movements. Interestingly, eye movements in our experiment did not represent the frequency value of each animal word per se (e.g., low frequency to the left, high frequency to the right), rather the similarity in the frequency space (same frequency value to the left, different frequency value to the right). We propose at least two interpretations of this finding. First, this can be potentially interpreted as a magnitude code (76), with small differences mapped to the left, and large differences to the right, accordingly with the well-documented propensity to map small and big quantities on the left–right space (SNARC-like effects, ref. 31). Since the category of animals, contrary to that of numbers, does not have a predefined order, what is mapped on the left–right continuum is the magnitude of each transition along the most salient lexical–semantic dimension (i.e., frequency). A possible alternative explanation for the reported effect, however, can be formulated under a cognitive control framework: Participants might have a bias toward looking to the left when they stay in the same “state” or maintain the same search strategy (e.g., “keep mentioning frequent animals” or “keep mentioning atypical animals”), switching to the right of the visual field when they change the strategy (e.g., “now mention an atypical/frequent animal”). Why (not) changing the current strategy should be associated with the right (left), however, remains unclear. More experiments are required to adjudicate between these alternatives. However, it is worth mentioning that the cognitive control interpretation would still be in accordance with a transition magnitude code: smaller effort to remain in the same strategy mapped to the left vs. bigger effort to change the current strategy mapped to the right. More speculatively, low-dimensional geometries such as lines (typical of magnitudes and numbers) and rings (typical of colors) might provide basis sets or primitives for scaffolding higher dimensional conceptual spaces, and this might be reflected in gaze behavior.

Before concluding, It worth asking why the reported effects were not ubiquitous to all the participants, especially when tested with single-subject analyses. In this experiment, we did not explore under what specific circumstances and manipulations the reported effects are observable, whether task constraints inherently trigger the spontaneous use of an oculomotor representational strategy, or whether individual differences exist across participants that might correlate with internal representational strategies in their memory. Further tailored experiments should address more carefully all these aspects and test how generalizable our results are to everyday situations and/or to all individuals.

To conclude, we have provided preliminary evidence that spontaneous eye movements might reflect the representational geometry of mentally navigated conceptual spaces, thus linking oculomotor behavior to cognitive mapping. These results could pave the way to a thorough investigation of conceptual spaces through gaze behavior.

## Methods

### Participants.

Thirty Italian volunteers participated in the experiment (mean age = 24.3, SD = 3.2, 18 females). They all gave informed consent and were reimbursed for their time with 7 euros/h. All had normal vision, no history of neurological disease, and were right-handed. For the number condition, one participant was discarded for not pronouncing the number “eleven.” For the color condition, two participants were discarded for not pronouncing the colors “lilac” and “turquoise.” Sample size was determined without an a priori power analysis, due to the lack of previous studies investigating eye movements and cognitive maps, but was consistent with G*Power sample size estimation at 85% power, alpha = 0.05 and assumed medium effect size of d = 0.5, which resulted in n = 27 participants. The study was approved by the Ethical Committee of the University of Trento (Comitato Etico per la Ricerca).

### Stimuli.

As the experiment required participants to freely and spontaneously generate words, no specific stimulation was provided. For the color condition, participants were instructed to memorize and then retrieve 12 colors: red, orange, yellow, lime, green, turquoise, cyan, blue, violet, lilac, pink, and magenta (in Italian: rosso, arancione, giallo, lime, verde, turchese, azzurro, blu, viola, lilla, rosa, and magenta). Color names were presented on the day before the experiment in association with color patches, which hues were selected from the internet. As the 12 colors used were quite common in the Italian language, no further specific validation of these stimuli was performed.

### Training Tasks for Colors.

On the day before the eye-tracking experiment participants were sent a link to a training phase, to be performed remotely via Pavlovia (https://pavlovia.org). The training consisted of three tasks. During the Encoding task (task 1), participants were presented with the 12 color names next to the corresponding 12 color patches, and they had to passively view and memorize them (*SI Appendix*, Fig. S4*A*). They could proceed to the next color by pressing a key on their keyboard. Each color was presented twice, in random order. During the Test task (task 2) we presented each color name next to a pseudorandomly chosen color patch, and participants were supposed to answer Yes or No as to whether the patch matched the color name (*SI Appendix*, Fig. S4*B*). Finally, during the Similarity Judgment task (task 3), participants were presented with two color names next to each other and they were asked to rate their similarity on a scale from 1 to 9, where 1 = low similarity and 9 = high similarity. Each color pair was presented twice, reversing the order of the two colors across repetitions. The entire training phase lasted about 30 min and was repeated identically on the day of the eye-tracking experiment, to ensure that participants remembered the color space to forage concepts from.

### Similarity Ratings and MDS-Reconstructed Color Space.

The similarity judgments between colors, collected across the two days, were averaged and used to construct a dissimilarity matrix. Following Shepard and Cooper (40), we then applied multidimensional scaling (MDS, using the MATLAB function *cmdscale*) to recover the bidimensional reconstruction of the color space (color wheel) for each individual participant. Euclidean distances between color points in these subject-specific color spaces were used for our correlational analyses with eye movements.

### Experimental Setup and Eye-Tracking Acquisition.

Participants sat in front of a monitor at a distance of approximately 60 cm with their head resting on a chin rest (readapted to be used on the forehead, to prevent excessive vertical movements when speaking). We continuously recorded gaze at a sampling rate of 1,000 Hz using an EyeLink 1000 Plus (SR Research). Before each block, we calibrated the eye tracker using the built-in calibration and validation protocols from the EyeLink software. Participants had earphones through which they could listen to sounds indicating the beginning and end of each block, as well as the metronome (see below: Main task). The lights in the room were switched off and the monitor was covered with a slightly larger white wooden panel to block any unwanted source of light or visual influence from it.

### Main Task.

After the eye-tracking calibration, participants read on the screen the category of concepts they had to think about on the screen. The monitor was then covered with the wooden panel and the experimenter started the block: This was signaled by a quick alerting sound that participants could hear from the earphones, and that was followed by a MATLAB-generated metronome at the frequency of 0.75 Hz. Although Loetscher et al. (32) reported using a frequency of 1 Hz, we opted for a slightly slower rhythm after some piloting without eye tracking on our colleagues, where they experienced the pace as too fast. Each block lasted 2 min, and participants were notified that the block ended via a second sound. There were three blocks for each category (numbers, colors, animals). Participants’ voice was recorded using custom Psychtoolbox functions in the MATLAB environment, via the built-in microphone of the earphones used to present sounds. As a cover story, participants were told they participated in an experiment designed to investigate how word generation is affected by having the eyes open vs. closed. They were told to be part of the “eyes open” group, and for that reason, they had to limit blinking. They were also told to not fixate on anything specific in front of them, but just to avoid looking around too much because otherwise the eye-tracker could lose the signal. There was no “eyes closed” group, and participants were debriefed after the end of the experiment.

### Audio Segmentation.

We segmented the audio tracks for each participant and block using a combination of custom MATLAB scripts, PRAAT, and Audacity. For each participant and block, we had a transcript of each pronounced word with the associated onset and offset.

### Eye-Tracking Analysis.

Eye-tracking data were converted from edf to MATLAB using the Edf2Mat toolbox (https://github.com/uzh/edf-converter). We excluded from the time series the time points where the eye-tracking signal was not available, due to blinks or because participants looked beyond the field of view of the eye-tracker, as automatically detected by the Eyelink. We did not apply any further preprocessing, inspired by van Ede et al. (61), who reported that their hypothesized gaze attentional biases were detected already before signal correction and because we hypothesized the predicted effect should emerge at the level of raw, spontaneous, gaze behavior. Then, following the approach by Loetscher et al. (32), we extracted the median gaze position in the 500 ms window before each word was pronounced. The median was chosen because it is more robust to outliers. This information was then used to either compute the trial-by-trial change in gaze coordinates, necessary for our main correlational analysis (Fig. 1*C*, see for instance results in Fig. 2*A* or Fig. 3*C*), or to compute the average “absolute” gaze position when a specific concept word was mentioned, to reconstruct the “visualized” conceptual spaces as for instance in Fig. 2 *C* and *D* or Fig. 3 *E* and *F*.

### Leave-One-Out Cross-Validated (LOOCV) LDA.

To attempt a classification of numbers (small vs. large, see main text) or of colors (12 colors, see main text) at the single subject level, we applied a LOOCV or leave-one-trial-out LDA. We have used the LDA classifier implemented in the CoSMoMVPA toolbox for Matlab. In the number condition, we first extracted the horizontal X coordinates in the visual space associated with each trial (mentioned number). Next, we recodified each trial as “1” (small) if the mentioned number was smaller or equal to 6, as “2” if the mentioned number was equal or larger to 7. Using Matlab 2022b, we created a partitioning scheme creating N folds where N is the number of trials. For each fold, all the trials minus one (the “left out”) serves as a Training Set, while the “left out” trial serves as a Test Set. In each Training Set, the number of observations for the two classes (small vs. big, that is 1 vs. 2) is likely to be unbalanced, therefore we applied a random resampling procedure to ensure an equal distribution of the two classes in the Training Set, using Matlab function “randsample”. We applied the CoSMoMVPa function “cosmo_classify_lda” to obtain a prediction of the class of the Test Set. We computed the “Observed Accuracy” for each subject by calculating the proportion of correct predictions. We repeated this procedure 1,000 times by randomly shuffling the class labels, computing 1,000 values of “Permuted Accuracy.” This resulted in a null-distribution of surrogate accuracies that allowed us to answer to the question of how likely it was to obtain the Observed Accuracy for each subject under the (null) hypothesis of random assignment of visual coordinates to numerical conditions. We calculated the probability (*P*) value as the mean number of Permuted Accuracies equal to or greater than the Observed Accuracy.

In the case of colors, we used the same exact procedure outlined above for the “Number” condition, with the major difference here that the binary classification was performed with bidimensional coordinates for all the possible pairwise combinations between colors—a necessary step to verify whether accuracy scales as a function of distance in color space. This means that for each subject we had a 12 × 12 accuracy matrix, with each cell representing the accuracy of the LDA for that specific color pair. The overall observed accuracy was computed as the average of the lower off-diagonal triangle of this symmetrized matrix, and the significance of the observed result was tested using the same permutation approach used for the “Number” conditions. Contrary to the number condition where we could classify small vs. large numbers, in the color condition, we needed to test all pairwise combinations, thus diminishing drastically the number of datapoints used for the cross-validated procedure in our classifier: Attempting this in our original dataset indeed resulted in overfitted responses, with the classifier performing above chance on the Training Set (avg. accuracy > 55%) and below chance in the Test Set. We thus analyzed data coming from a different dataset composed of five individuals that were tested multiple times to increase the number of datapoints, following the approach of precision neuroimaging (e.g., see refs. 42 and 43). These individuals performed the color foraging task with the same 12 colors of Experiment 1, for eight blocks per day, for 3 d in a row, resulting in 24 blocks (compared to only three in Experiment 1) and more than 2,000 trials/observations per subject. This dataset was collected not only as part of our decoding attempt, but also as part of a follow-up pilot experiment where participants had to also forage for “emotion” names, in different blocks. The “emotion” condition (that in this case substituted the “number” condition) is not relevant for the present work and will thus be ignored (there was no task or any kind of influence between emotion and color condition before the eye-tracking experiment). Crucially, we implemented a second, simple but important manipulation in the color condition: Participants were not asked the similarity judgments between colors before the experiment, but they were simply presented acoustically with a list of the same 12 colors of Experiment 1, via an online procedure the day before the experiment, asking them to listen to the color names (presented in random order) for multiple times, without completing any task. On the day of the experiment, the researcher asked the participants to repeat the color names, to make sure they were automatically memorized.

### Animal Words and Their Distances.

As a proxy of distance between animal words, we used several approaches that were used in previous studies to investigate semantic representations (see *Results* for references). First, we used pretrained linguistic vectors from FastText, freely available from the website https://fasttext.cc. Distances between words in the full 300-dimensional space were computed using MATLAB built-in cosine distance (Fig. 4 *A*, *Left*). Second, we coded distances among subsequently pronounced animals as 0 or 1 based on whether whey implied a change in cluster or not (Fig. 4 *A*, *Mid-Left*), following the labeling of Troyer et al. (49), Hills et al. (50), and Zemla et al. (51) among others. Following these previous studies, we considered a passage to a word that does not share any semantic label with the previous one as jump to a new cluster (coded as 1 in an otherwise 0s vector). A similar analysis where instead of 1 or 0 we put the exact number of shared labels was conducted but yielded almost identical results, and it was therefore not reported for readability of the manuscript. Third, we modeled distances as the inverse of similarity (from 1 to 9), as rated by chatGPT (Fig. 4 *A*, *Mid Right*; see next section for details). We also performed Principal Component Analysis using MATLAB built-in *pca* function, and Euclidean distances along PC1 were considered (Fig. 4 *A*, *Right*). Finally, we modeled distances as differences in word frequency, which was extracted from the freely available database by Crepaldi et al. (56) Subtlex-it (https://osf.io/zg7sc/): For each word, the corresponding frequency value was extracted and the distance between two words was computed as the unsigned difference between the two.

### Modeling Distances with chatGPT.

For each animal sequence (per participant and block) of length *n* animals, we created *n*−1 animal pairs, pairing each word with the previous and the subsequent one. We asked chatGPT (https://chat.openai.com/chat, by OpenAI) to evaluate the similarity from 1 to 9 between the two animals of each pair (*SI Appendix*, Fig. S10). The range 1 to 9 was chosen to match that used for colors. This resulted in a vector of similarity judgments per participant and per block. This procedure was repeated twice for each animal pair to increase reliability, and the two results were averaged. The similarity vector was then transformed into a dissimilarity/distance vector simply by inverting the scores (10 minus similarity).

In order to increase the stability and reliability of these judgments we constructed a matrix of distances between animals where we put, for each animal pair, the average of the similarity scores that chatGPT gave across all the animal lists. This led to a big matrix of size ~310 × 310 (where 310 are the unique animals that have been mentioned across the whole experiment, so across participants and blocks), and we used this matrix as source of semantic distances, recreating the trial-by-trial distance vector and running the correlation analysis. Similarity ratings significantly correlated with cosine distance between the 300-dimensional linguistic vectors of FastText (*P* < 0.001), supporting the robustness and the reliability of the approach.

### Statistical Analyses.

Statistical analyses were performed using a combination of Null-Hypothesis Statistical Testing (NHST) and Bayesian statistics. We used custom MATLAB scripts and the software JASP (https://jasp-stats.org/).

## Supplementary Material

Appendix 01 (PDF)

## Data Availability

Data and code to generate the main results and figures are available at DOI: 10.17605/OSF.IO/9GXBE (77).
