# Supplementary material for: Spontaneous eye movements reflect the representational geometries of conceptual spaces
Source: Proc Natl Acad Sci U S A. 2024 Apr 18;121(17):e2403858121. doi: 10.1073/pnas.2403858121 (PMC11046636; doi:10.1073/pnas.2403858121)
Supplement: Supplementary file 1 — Appendix 01 (PDF) [file pnas.2403858121.sapp.pdf]

Spontaneous eye movements reflect the representational geometries of conceptual spaces

Simone Viganò[1,2,†], Rena Bayramova[1,3], Christian F. Doeller[1,4,5,6*], Roberto Bottini[2,*,†]

[1] Max Planck Institute for Human Cognitive and Brain Sciences, Leipzig, Germany
[2] Center for Mind/Brain Sciences, University of Trento, Rovereto, Italy
[3] Max Planck School of Cognition, Max Planck Institute of Human Cognitive and Brain Sciences, Leipzig, Germany
[4] Kavli Institute for Systems Neuroscience, Center for Neural Computation, The Egil and Pauline Braathen and Fred Kavli Center for Cortical Microcircuits, Jebsen Center for Alzheimer's Disease, Norwegian University of Science and Technology, Trondheim, Norway
[5] Wilhelm Wundt Institute of Psychology, Leipzig University, Leipzig, Germany.
[6] Department of Psychology, Technische Universität Dresden, Dresden, Germany

[*] co-senior authors
† corresponding authors: S Viganò vigano@cbs.mpg.de; R Bottini roberto.bottini@unitn.it

Supplementary Materials

Supplementary Figure 1
Supplementary Figure 2
Supplementary Figure 3
Supplementary Figure 4
Supplementary Figure 5
Supplementary Figure 6
Supplementary Figure 7
Supplementary Figure 8
Supplementary Figure 9
Supplementary Figure 10
Supplementary Figure 11
Supplementary Figure 12

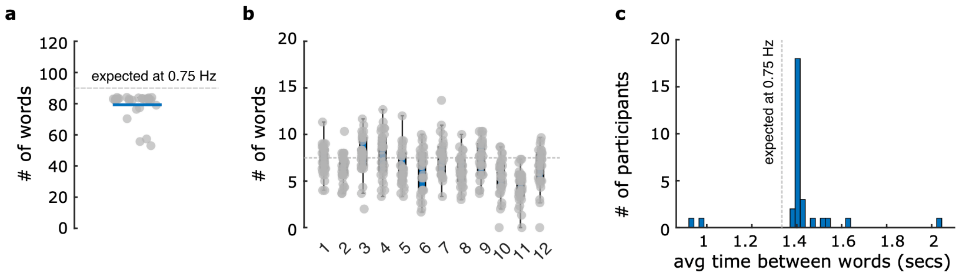

Supplementary Figure 1 - a, number of words produced by each participant in the number condition. The horizontal blue bar represents the group mean. The horizontal grey line represents the expected number of words if participants were perfectly following the rhythm of the metronome. **b**, same as a, but divided for each number word. **c**, average time between pronounced words. The vertical grey line is the expected frequency.

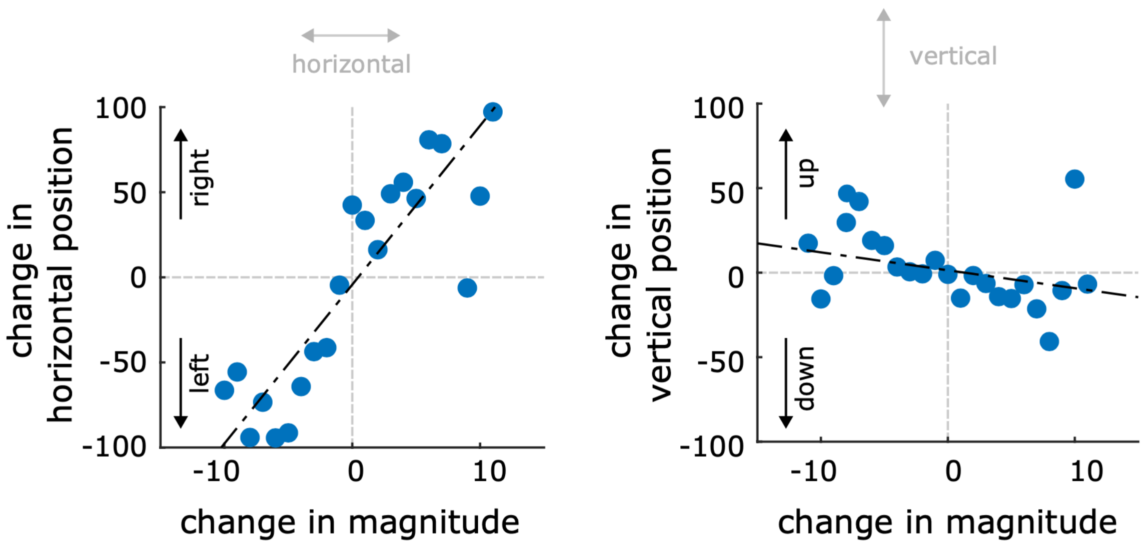

Supplementary Figure 2 - Visualization of the effect reported in Fig. 2a of the main text. Different levels of change in magnitude (signed) are plotted on the x-axis and are associated with different changes in either horizontal (left) or vertical (right) gaze position. Each dot is the average across subjects and trials responding to that particular change in magnitude. For instance, a change in magnitude of +5 could have originated from the transition 2→7 as well as from the transition 7→12.

a

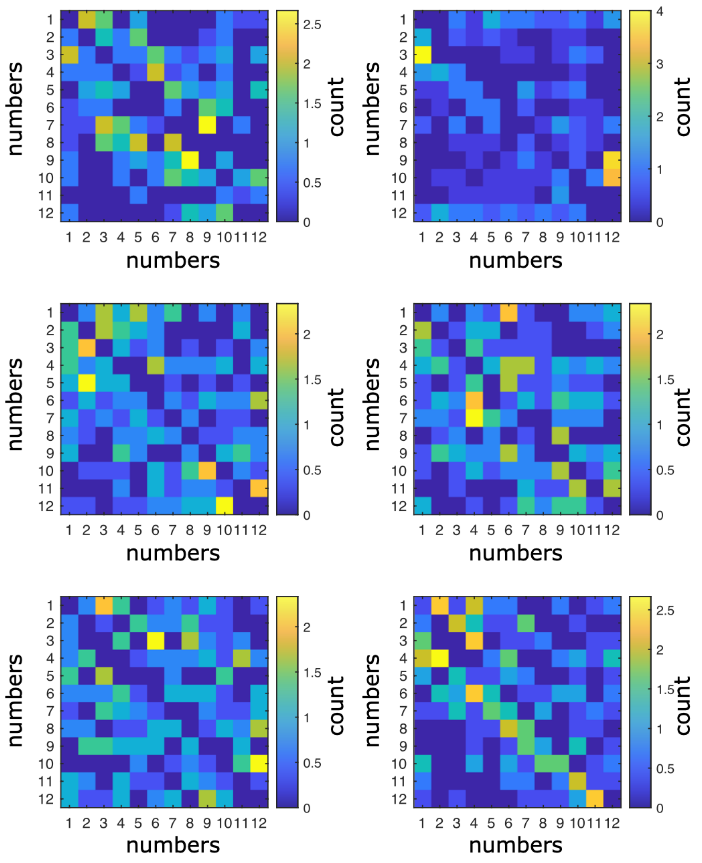

Supplementary Figure 3 - Transition frequencies from one number to another in the number generation task for 6 subjects. Each matrix entry indicates how many times, on average across the 3 blocks, that participant mentioned a number (e.g., “7”, x-axis) after another one (e.g., “3”, y-axis). Eye movements did not reflect these transition probabilities (mean ρ = 0.0004, p =.97).

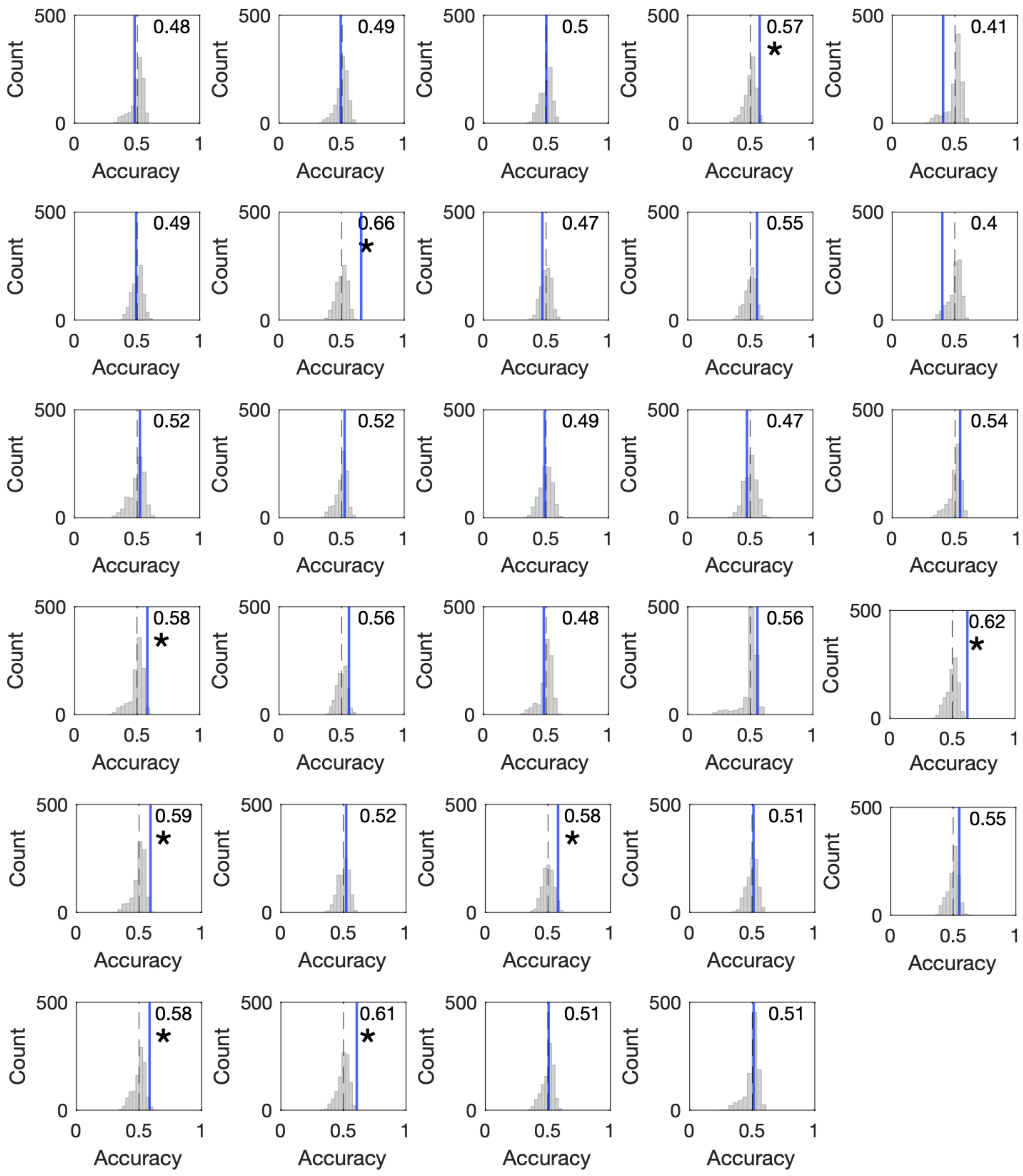

Supplementary Figure 4 - We attempted classification of small (1 to 6) vs large (7 to 12) numbers using a leave-one-out cross validated scheme and a linear discriminant analysis (LDA) on each individual participant's horizontal gaze coordinates (see Methods). Each panel is a subject, the vertical dashed line is the theoretical chance level at 50% of accuracy, the grey distribution is an histogram summarising the accuracies obtained by 1000 iterations of the analysis with random permutation of class labels. The blue line indicates the observed real accuracy of the classifier when labels were not permuted. Its value is also reported within the panel. The asterisks indicate that the probability of observing such accuracy under the distribution of surrogate values is < .05.

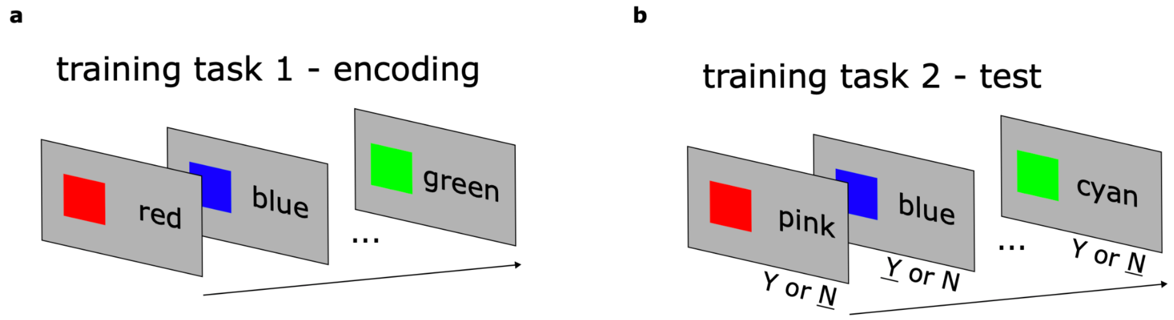

Supplementary Figure 5 - a, Participants are first passively presented with colour patches and their corresponding colour names (task 1). **b,** Then, they are presented again with colour patches, but now with pseudorandom colour labels next to them, for which they have to evaluate the correctness of the association (task 2).

a

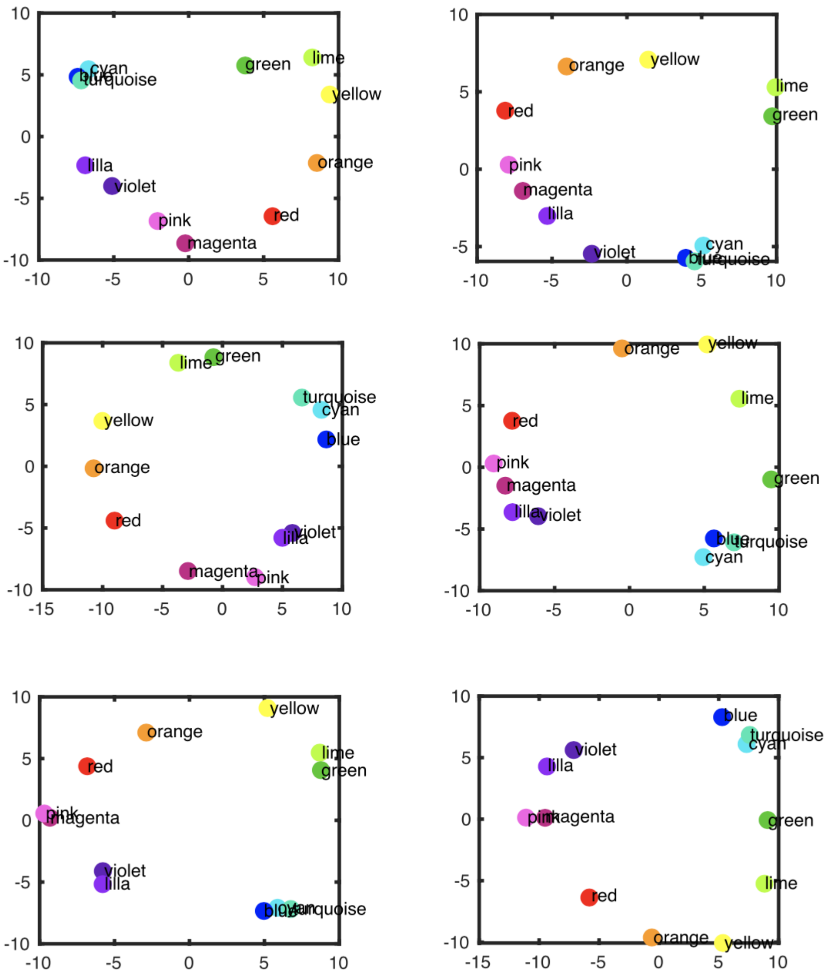

Supplementary Figure 6 - a, other examples of colour wheels reconstructed using MDS for 6 additional subjects

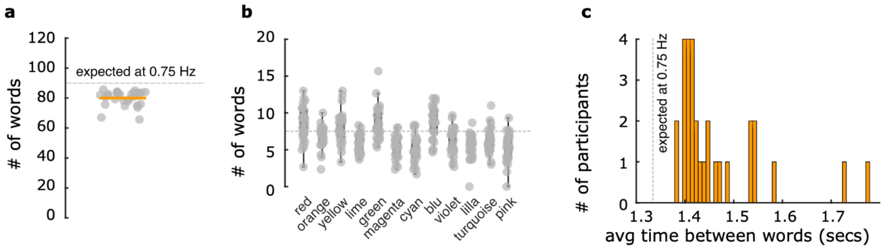

Supplementary Figure 7 - a, number of words produced by each participant in the colour condition. The horizontal orange bar represents the group mean. The horizontal grey line represents the expected number of words if participants were perfectly following the rhythm of the metronome. **b**, same as a, but divided for each colour word. **c**, average time between pronounced colour words. The vertical grey line is the expected frequency.

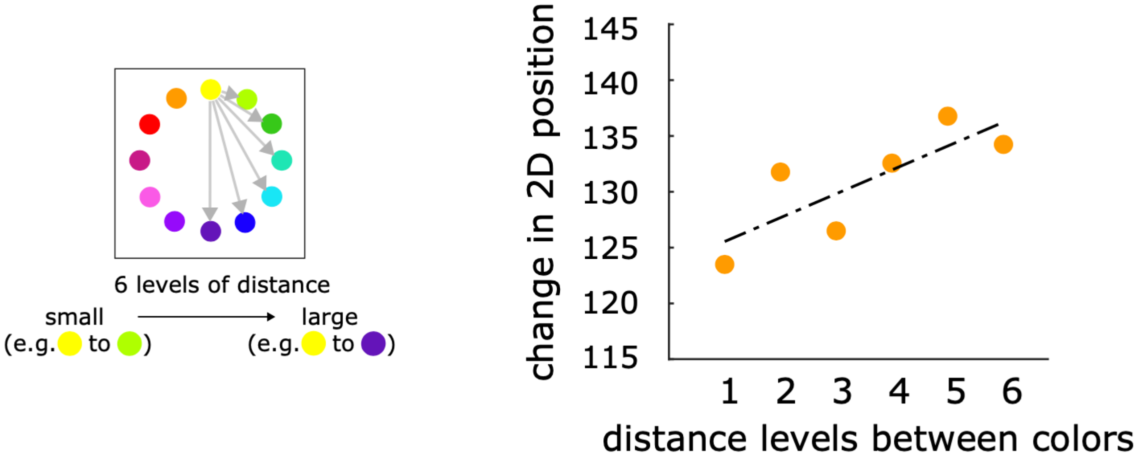

Supplementary Figure 8 - Tentative visualisation of the effect reported in Fig. 3c of the main text, following the same logics of Supplementary Figure 2, but for colours. For pure visualisation, we considered 6 possible levels of distances from each point of a perfect circle of 12 colours to every other colour. We thus divided for each participant the distance values between colours in 6 equal groups of increasing level (that is, distance level 1 contains the 1/6th shortest distances for each participant) and we extracted the average change in 2D position for the corresponding trials.

a

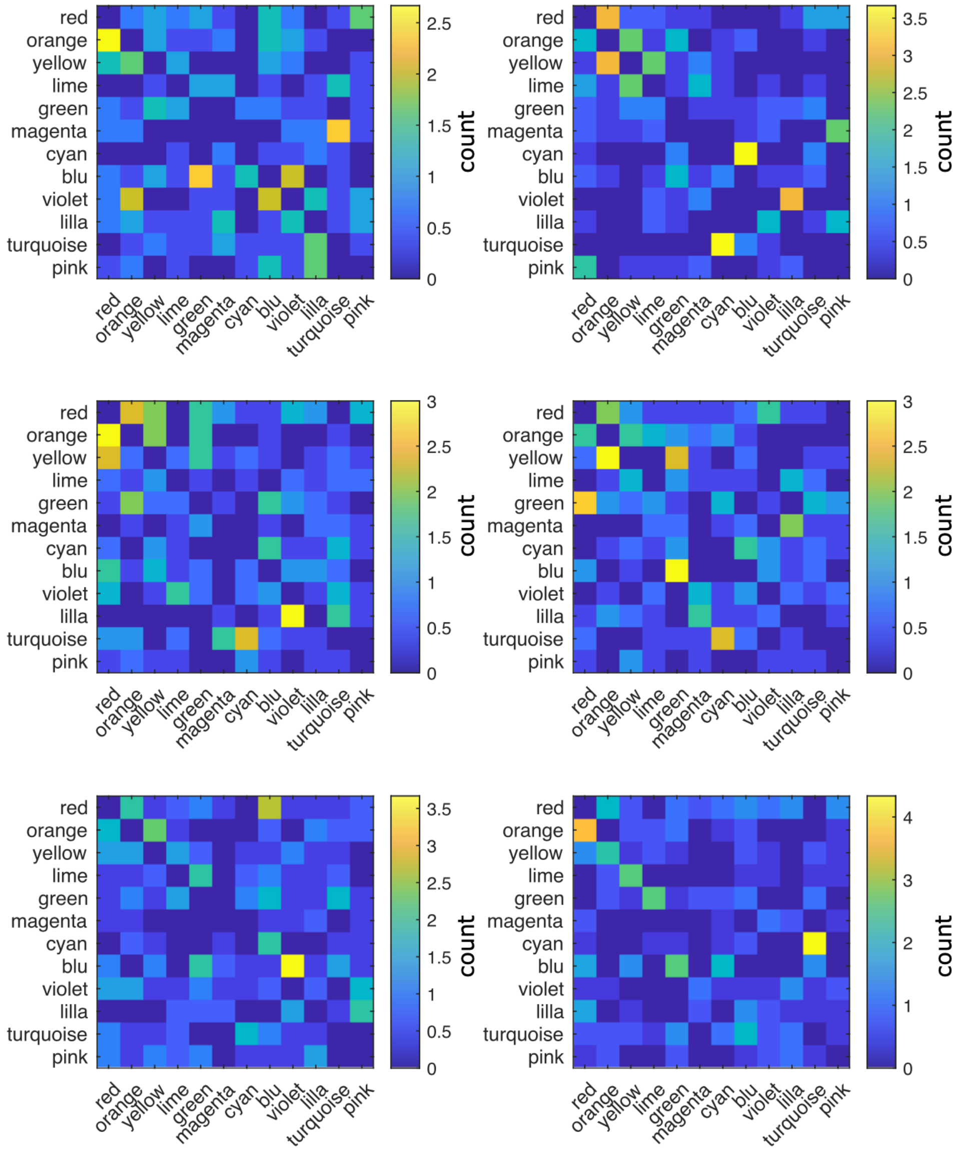

Supplementary Figure 9 - Transition frequencies from one colour to another in the colour generation task for 6 subjects. Each matrix entry indicates how many times, on average across the 3 blocks, that participant mentioned a colour (e.g., “violet”, x-axis) after another one (e.g., “red”, y-axis). Eye movements did not reflect these transition probabilities (mean ρ = -0.02, p =.19).

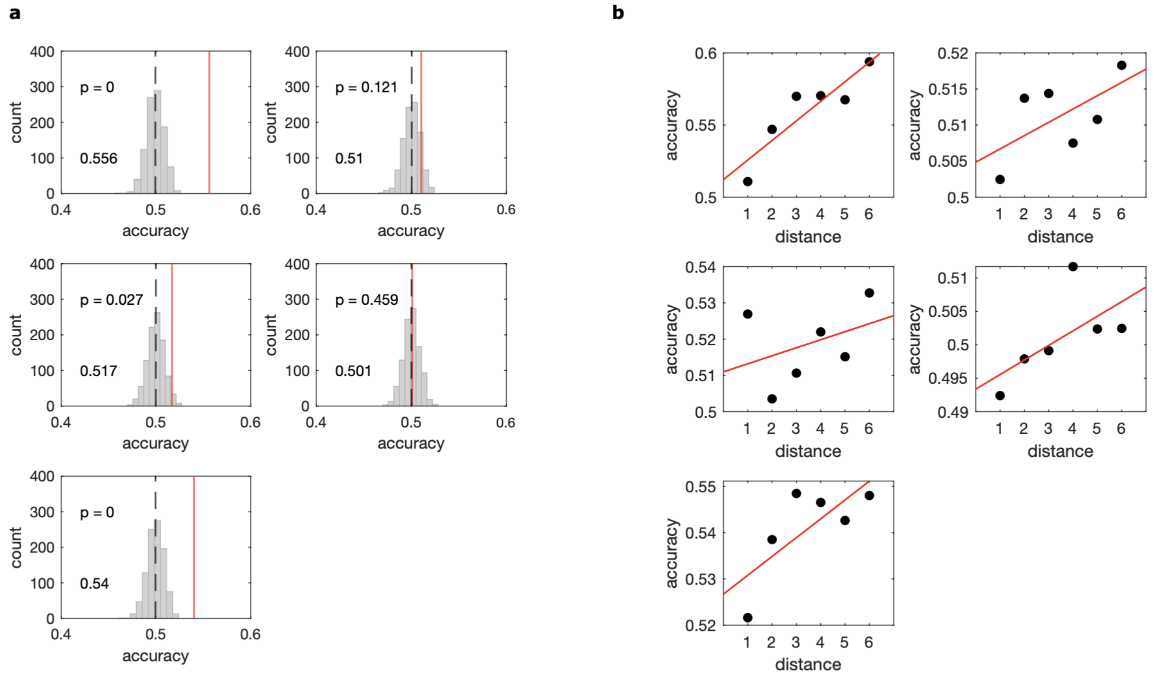

Supplementary Figure 10 - a, we attempted single trial classification of colours using a leave-one-out cross validated scheme and a LDA classifier on a small sample of densely tested subjects (see Methods) and their bidimensional gaze coordinates. Each panel is a subject, the dashed black vertical line indicates the theoretical chance level at 50%, the grey bar histogram indicates the distribution of surrogates' accuracies obtained by repeating the analysis 1000 times with random shuffling of class labels. The red vertical line indicates the real observed accuracy of the classifier when labels were not shuffled, and its value is also reported numerically, together with the probability (p) value of obtaining such a score under the distribution of surrogate values. b, shows for each individual subject the accuracy of the classifier as a function of the distance between colours. A positive trend of the fitted line indicates that colours that are similar to each other are more difficult to classify apart, while those that are more dissimilar are more easily distinguishable using gaze coordinates, resembling the pattern of Supplementary Figure 8.

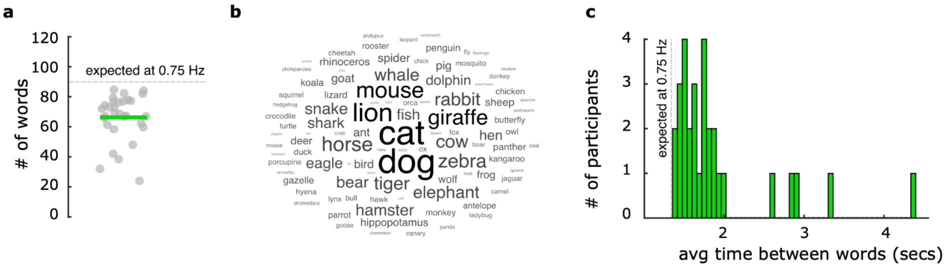

Supplementary Figure 11 - a, number of words produced by each participant in the animal condition. The horizontal green bar represents the group mean. The horizontal grey line represents the expected number of words if participants were perfectly following the rhythm of the metronome. **b**, wordcloud visualisation of the most frequent words pronounced by participants. **c**, average time between pronounced animal words. The vertical grey line is the expected frequency

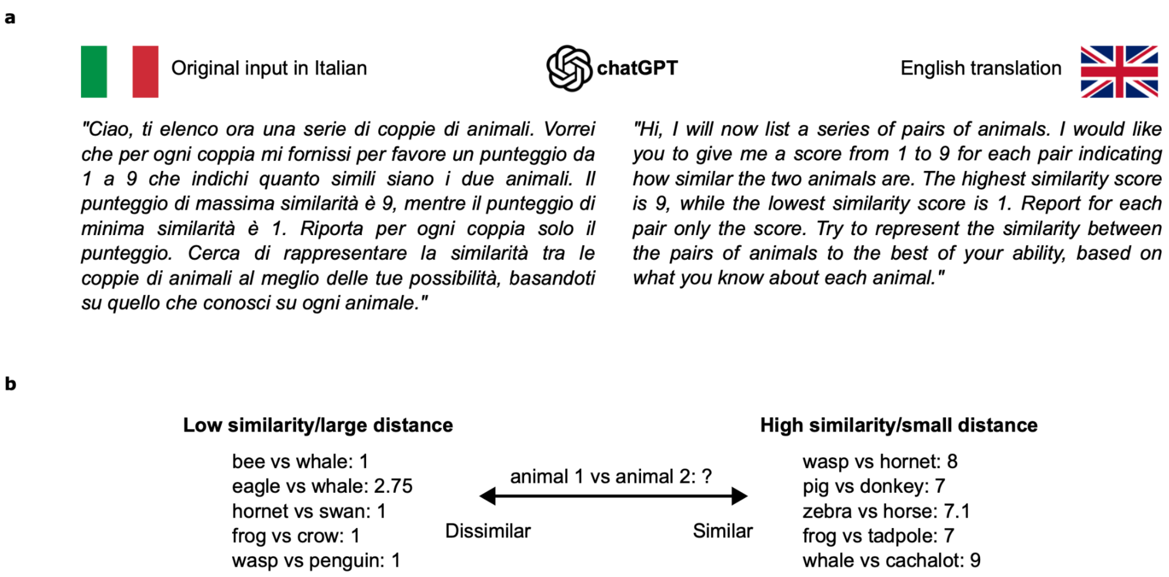

Supplementary Figure 12 - a, Input provided to chatGPT to evaluate the similarity between animal names (see Methods). **b**, Examples of highly similar (similarity scores closer to 9) or highly different (similarity scores closer to 1) animals as judged by chatGPT. The similarity score was later transformed to a distance metric by computing its inverse.
